# Pharmacy Workplace Wellbeing and Resilience: Themes Identified from a Hermeneutic Phenomenological Analysis with Future Recommendations

**DOI:** 10.3390/pharmacy10060158

**Published:** 2022-11-23

**Authors:** Jon C. Schommer, Caroline A. Gaither, Nancy A. Alvarez, SuHak Lee, April M. Shaughnessy, Vibhuti Arya, Lourdes G. Planas, Olajide Fadare, Matthew J. Witry

**Affiliations:** 1 Department of Pharmaceutical Care & Health Systems (PCHS), College of Pharmacy, University of Minnesota, 308 Harvard Street SE, Minneapolis, MN 55455, USA; 2 R. Ken Coit College of Pharmacy–Phoenix, University of Arizona, 650 East Van Buren Street, Phoenix, AZ 85004, USA; 3 American Pharmacists Association, 2215 Constitution Avenue NW, Washington, DC 20037, USA; 4 College of Pharmacy and Health Sciences, St. John’s University, 8000 Utopia Parkway, Queens, New York, NY 11439, USA; 5 College of Pharmacy, University of Oklahoma, 1110 N Stonewall, Room 243, Oklahoma City, OK 73117, USA; 6 College of Pharmacy, University of Iowa, 180 South Grand Avenue, Iowa City, IA 52242, USA

**Keywords:** pharmacist, technician, pharmacy, stress, burnout, distress, moral injury, workism, trauma, responsibility, autonomy, subjection, sociocultural, systems, hermeneutic phenomenology

## Abstract

This study applied a hermeneutic phenomenological approach to better understand pharmacy workplace wellbeing and resilience using respondents’ written comments along with a blend of the researchers’ understanding of the phenomenon and the published literature. Our goal was to apply this understanding to recommendations for the pharmacy workforce and corresponding future research. Data were obtained from the 2021 APhA/NASPA National State-Based Pharmacy Workplace Survey, launched in the United States in April 2021. Promotion of the online survey to pharmacy personnel was accomplished through social media, email, and online periodicals. Responses continued to be received through the end of 2021. A data file containing 6973 responses was downloaded on 7 January 2022 for analysis. Usable responses were from those who wrote an in-depth comment detailing stories and experiences related to pharmacy workplace and resilience. There were 614 respondents who wrote such comments. The findings revealed that business models driven by mechanized assembly line processes, business metrics that supersede patient outcomes, and reduction of pharmacy personnel’s professional judgement have contributed to the decline in the experience of providing patient care in today’s health systems. The portrait of respondents’ lived experiences regarding pharmacy workplace wellbeing and resilience was beyond the individual level and revealed the need for systems change. We propose several areas for expanded inquiry in this domain: (1) shared trauma, (2) professional responsibility and autonomy, (3) learned subjection, (4) moral injury and moral distress, (5) sociocultural effects, and (6) health systems change.

## 1. Introduction

### 1.1. Pharmacy Workplace Wellbeing and Resilience

Pharmacy personnel’s workplace issues and their relationship to personal wellbeing continue to be critical, complex issues across all practice settings [1], and have been further exacerbated and exposed under the COVID-19 pandemic [2,3,4,5,6,7]. In addition to burnout, stressful pharmacy job demands have been linked to patient safety concerns, especially medication errors [8,9].

Even before the COVID-19 pandemic, pharmacy personnel in the United States were experiencing high levels of stress due to the U.S. health care system’s shift from a public service to a business model that began in the latter half of the 20th century [10]. Pharmacy practice involves intimate caregiving relationships that often require the suspension of routine pharmacy operations in order to address serious patient-specific needs [9,10]. Business models driven by mechanized assembly line processes, business metrics that supersede patient outcomes, and reduction of pharmacy personnel’s professional judgement have contributed to the decline in the experience of providing patient care in today’s health systems [10,11,12].

Traditional approaches for addressing stress and burnout are to (1) identify them using sound measures and (2) build personal resilience as a key requisite for coping. Regarding identification and measurement, useful models of job stress and burnout have been developed and applied for identifying factors that cause stress, the outcomes of such stress, and ways to manage and prevent stress [3,4,5,6,7,13,14,15,16]. A recent integrative review of burnout and stress literature in pharmacy identified 491 articles that used 11 psychometrically sound measures [15]. Regarding personal resilience, it is the most often identified way to cope with stress [12,15,17,18]. In addition to a focus on individual responsibility for resilience, more expansive stress management and prevention literature is beginning to emerge in pharmacy [19]. For example, recent research in pharmacy [9,11] suggests that the COVID-19 pandemic was a shared crisis that extended beyond individual and organization levels, to the health care system itself, and in some cases, at a sociocultural level [9,11,12]. Thus, ways to cope with stress may have broadened as well from the individual and organizational level to health systems and societal levels. Some examples of these are presented next.

### 1.2. Health Systems and Moral Distress or Moral Injury

The U.S. health care system’s shift from a public service to a business model [10] pressured pharmacy to meet efficiency challenges through market power dynamics, negotiated contracts, pay-for-performance incentives, and corporate-level metrics. However, top-down, rigid, business-centric management decisions created dissonance for pharmacy personnel’s ability to provide patient-centered care, exercise professional judgement, and experience joy and meaning in providing care [9,11,12]. Two phenomena, moral distress and moral injury, may be useful to understand the dissonance expressed by some in their daily work. Moral distress is a “psychological disequilibrium which occurs when a provider is able to make a moral judgment about the correct choice, but is not able to provide the care that is perceived to be ‘right’ or ‘best’ for the patient” [20]. As Talbot and Dean [21] wrote about developing moral injury:

*“Navigating an ethical path among such intensely competing drivers is emotionally and morally exhausting … Routinely experiencing the suffering, anguish, and loss of being unable to deliver the care that patients need is deeply painful. These routine, incessant betrayals of patient care and trust are examples of ‘death by a thousand cuts.’ Any one of them, delivered alone, might heal. But repeated on a daily basis, they coalesce into the moral injury of health care.”*

These health system effects already were detected before the COVID-19 pandemic [1,12], but the onset of the pandemic exposed and amplified the likely experiences of moral distress and moral injury for pharmacy personnel.

### 1.3. The Sociocultural Phenomenon of Workism

In addition, a sociocultural phenomenon called ‘workism’ has affected pharmacy personnel wellbeing and resilience [12,22,23] by building an expectation that work is the centerpiece of one’s identity and life’s purpose. Thompson states that “a culture that funnels its dreams of self-actualization into salaried jobs is setting itself up for a collective anxiety, mass disappointment, and inevitable burnout” [22]. The majority of pharmacists in the United States are now under the age of 40 with less than 15 years of experience after licensure [1]. This group reports higher stress and burnout than those with more experience [1,5,14]. They passed through a childhood of extracurricular achievement, were told that their work should be their passion, took on high levels of student debt, and face the disturbance of social media which amplifies the pressure to project a superficial image of success to others [22]. Further, social media use is linked to dehumanization, anxiety, stress, and relational problems [23]. These sociocultural effects already were detected before the COVID-19 pandemic [1,12], but the onset of the pandemic exposed and amplified the notion of workism for pharmacy personnel.

### 1.4. Application of a Hermeneutic Phenomenological Approach

As we completed research in the pharmacy workplace and resilience domain, aspects of moral injury and workism emerged in the findings [9,11,12]. This led us to consider that relying on personal resilience as a key requisite for coping with stress and burnout is not sufficient and may actually harm individuals from thinking they are at sole fault for experiencing burnout. Health system and sociocultural effects were reported as contributors to stress, burnout, and feelings of despair since resilience could not be achieved within the situations being faced. In the 2021 American Pharmacists Association (APhA) and National Alliance of State Pharmacy Associations (NASPA) National State-Based Pharmacy Workplace Survey, a series of five open-ended questions was used to learn about respondents’ opinions and experiences in their own words. The first four questions focused on barriers and facilitators to their ability to perform duties for optimal patient care and/or ensuring patient safety. Findings from these questions have been published [9,11].

The fifth question simply asked if respondents had any other comments. We were surprised to find that 1327 (19%) out of 6973 responders took the time to write additional comments. Following guidance from phenomenology research [24,25,26,27,28,29], we included comments for analysis if the comments included information about the respondent’s “lived experience” [25,26]. Applying this approach resulted in 614 (46%) comments that were in-depth responses that described stories and experiences. The comments that did not meet our inclusion criteria (the other 54%) mostly related to thanking us for conducting the survey with some comments also giving suggestions about how to change pharmacy or healthcare. In order to gain a better understanding about workplace wellbeing and resilience, we turned to a hermeneutic phenomenological approach as a way to appreciate respondents’ lived experiences within their workplaces as described in their own words. This approach seeks to uncover meaning within the experience by analyzing narratives about the experience [24,25,26,27,28,29]. The 614 in-depth stories were well suited for such an approach.

### 1.5. Study Objectives

This study applied a hermeneutic phenomenological approach to better understand pharmacy workplace wellbeing and resilience. Rather than a focus on the causes of the phenomenon, our aim was to present a portrait of the respondents’ lived experiences. The objective of our inquiry was to describe pharmacy workplace wellbeing and resilience using respondents’ written comments along with a blend of the researchers’ understanding of the phenomenon and the published literature [28,29]. Our goal was to apply this understanding to recommendations for the pharmacy workforce and corresponding future research.

## 2. Materials and Methods

### 2.1. Data Source

Data were obtained from the 2021 APhA/NASPA National State-Based Pharmacy Workplace Survey [9], launched nationally in April 2021 by the two organizations. Promotion of the online survey to pharmacy personnel was accomplished through social media, email, and online periodicals. Responses continued to be received through the end of 2021. A data file containing 6973 responses was downloaded on 7 January 2022 for analysis.

For the purpose of this study, usable responses were from those who wrote an in-depth comment detailing stories and experiences related to pharmacy workplace wellbeing and resilience. There were 614 respondents who wrote such comments, and these were used for analysis.

### 2.2. Hermeneutic Phenomenological Analysis

The 614 in-depth comments were stored in a Word file and distributed to five research team members (NA, CG, SL, AS, JS). Written comments were read several times by each person independently. The main stories, anecdotes, and insights written by the respondents were analyzed through van Manen’s “inceptual process” of reflective wondering, deep questioning, attentive reminiscing, reflective epoché, and sensitively interpreting primal meanings of experiences [25,26]. We did this without imposing judgement and with self-reflexivity and recognition about our own experiences and beliefs. We focused on the respondents’ lived experiences and their ways of describing these experiences.

An example of this method is from a study of patients with depression [29]. Focusing on the causes of depression was deemed insufficient for understanding a portrait of the experience of depression itself. In that study, people described depression as a place that takes the person away from their “everydayness (alltaglichkeit) and their homelikeness (heimlichkeit)” to a place of “uncanniness and un-homelikeness”. Depression was described using figurative spatial imagery such as constriction, bottomless pit of darkness, wilderness or isolated places. We applied a similar approach to understanding a portrait of workplace wellbeing and resilience.

In our first step of analysis, we asked “What do the comments tell us about respondents’ lived experiences and how can they be described?” After this initial reading, the five team members met in March 2022 and identified issues like (1) desperation, (2) imagery of dying, (3) drowning, (4) moral injury, (5) hopelessness, and (6) regret. The initial consensus was that themes were consistent with the workplace burnout literature. After this meeting, each team member independently read the comments again and we reconvened in April 2022. During that same time, one team member (SL) conducted a literature review of the stress and burnout literature.

During the next team meeting, the same themes emerged, but discussion about the inability of pharmacy personnel to meet all of their expected responsibilities for their organization, profession and patients emerged. Comments were identified that went beyond the stress and burnout literature that focused on individual characteristics and rather, were about the overall health care system and sociocultural influences. This prompted the team to search the moral injury and workism literature, and also to meet with an expert in the area of pharmacist responsibilities (LP) for further discussion. This helped broaden the scope of descriptions into areas of responsibility, professionalism, health system effects, and sociocultural effects. During this meeting, the group expanded discussions into areas such as moral distress, learned helplessness, ‘metrics equal worth’, practice isolation, coaching/mentoring, perceived responsibility, just cultures, joy and meaning in work, and improving the experience of providing care.

In April 2022, one team member (NA) took the list of identified elements and began grouping lived experience exemplars for each element. As this was being completed, the other four team members consulted the literature and shared ideas with the whole team. In late April 2022, the team met again and reviewed the elements and exemplars that were identified. In May 2022, the team met to discuss elements and exemplars in order to reveal inceptual insights [25,26]. On June 16, 2022 the team members agreed upon 15 elements, with relevant exemplars and insights. At this point (June 2022), the team grouped the 15 elements into six (6) categories for review and discussion. These are described in the Appendix A (see Table A1).

In addition to the five research team members (NA, CG, SL, AS, JS), four additional research experts (VA, OF, LP, MW) were invited to review the elements and categories and develop recommendations for expanding pharmacy workforce wellbeing and resilience research. Each co-author focused on one of six categories:

1. Shared Trauma (VA)
2. Threats to Professional Responsibility and Autonomy (LP)
3. Learned Subjection (CG)
4. Moral Distress and Moral Injury (NA, AS)
5. Sociocultural Effects (SL, JS)
6. Plea for Health Systems Change (OF, MW)

### 2.3. Research Team and Reflexivity

As hermeneutic phenomenological analyses were completed, team member reflexivity was conducted so that assumptions were acknowledged and documented as part of the research process [30,31,32]. The research team for this paper consisted of nine people (NA, VA, OF, CG, SL, LP, AS, JS, MW). All nine have experience in pharmacist workforce and quality of work life research. Four members (NA, VA, SL, MW) hold PharmD degrees and have experience in advanced clinical care practice. Four members (CG, LP, JS, MW) hold PhD degrees in pharmacy. Four members (NA, VA, LP, AS) are actively engaged in legislative policy and advocacy work. All nine team members hold licenses to practice pharmacy. Each member of the team interacts with pharmacists and student pharmacists on a regular basis. Each member of the team has work experience in pharmacy practice and these experiences were shared during analytic discussions. During analysis, team members acknowledged how they were being affected by personal events that included learning about and supporting others through injustice, inequity, mental health, and dealing with suicide. Our team acknowledged how these events could impact our analysis and interpretation of the findings. The background of the research team provides strengths to this project that helped analyze and interpret the data collected. Personal presuppositions from both professional and personal experiences were noted and accounted for in how the analysis may have been influenced.

### 2.4. Rigor

Realism was supported by reaching congruence among research team members regarding the data‘s relevance, sense, and accuracy and the use of thick descriptions [31]. Credibility and authenticity were reinforced by multiple readings of the text, member checking, and documentation [27,31,32,33,34]. This helped assure that data interpretation echoed the respondents’ words and not the biases and viewpoints of the research team [27,31,32]. Audit trails were maintained in order to document each aspect of the research process [31,32].

## 3. Results

Fifteen elements emerged from the data that described pharmacy workplace wellbeing and resilience using respondents’ written comments along with a blend of the researchers’ understanding of the phenomenon and the published literature [28,29]. These 15 elements were grouped into six categories as follows:

1. Shared Trauma (Helplessness; De-professionalization of pharmacy)
2. Threats to Professional Responsibility and Autonomy (Disempowerment, Power-dependence)
3. Learned Subjection (Oppression; Abandonment; Depersonalization; Dehumanization)
4. Moral Distress and Moral Injury (Moral distress; Moral injury)
5. Sociocultural Effects (Despair; Disdain)
6. Plea for Health Systems Change (Different cultures; Now is the time; Plea for change)

Written exemplars for each element are presented in the Appendix A. It is noteworthy that the 15 elements and six categories are negative in nature. There were very few positive comments and these typically were about being thankful where they worked so that they could avoid one of the 15 elements we identified. The results show that the portrait of respondents’ lived experiences regarding pharmacy workplace wellbeing and resilience are beyond the individual level. There is shared malaise surrounding a collective experience of helplessness, loss of professional standing, oppression, moral injury, workism, and a plea for rescue. The results show the need for health care system and societal change and provide insights for future research. This will be presented in the discussion section of this paper.

## 4. Discussion

### 4.1. Limitations

Before the findings are discussed, several limitations should be considered. The results did not use a random sample of pharmacy personnel. Thus, the findings should be used for gaining insight and not be used for making estimates for or to generalize to the entire population of pharmacy personnel. Not all survey respondents provided written comments. It is likely that those who wrote comments had strong opinions or were interested in the topic. The majority of comments came from pharmacy personnel working in community practice. The distribution of respondents to the survey and associations between respondent types and patterns of response may be found in the full report [9].

### 4.2. Structure Used for the Discussion

The findings described pharmacy workplace wellbeing and resilience using survey respondents’ written comments along with a blend of the researchers’ understanding of the phenomenon and the published literature [28,29]. This portrait of the respondents’ lived experiences was used as a guide for us to develop recommendations for future research in this domain. The discussion is presented in six sections that describe proposed areas for future research.

### 4.3. Recommendations for Expansion of Research in Pharmacy Workplace Wellbeing and Resilience

#### 4.3.1. Shared Trauma

The experience of COVID-19, much like any other natural disaster or major event that communities experience collectively (e.g., hurricanes, 9/11/01 attacks in New York City, etc.) upended lives of millions and disrupted nearly every individual’s life across the globe. The extent of this disruption varied across communities, ranging from minor inconveniences to loss of lives, economic hardship, and long-term negative health consequences. Existing inequities were magnified, cracks across systems revealed in more pronounced manners, and impact on well-being and behavioral health emerged in a much stronger way across society. No matter where on the spectrum of disruption the individual experience of this trauma fell, communities of people were all collectively feeling the impact of this extraordinary situation. Particularly for healthcare professionals, this time has marked a shared trauma that both patients and their care providers were experiencing at the same time.

Shared trauma is “the affective, behavioral, cognitive, spiritual, and multi-modal responses that clinicians experience as a result of dual exposure to the same collective trauma as their clients” [35]. It is important to note that while persons providing care are impacted by the same trauma as patients, their response may not necessarily be the same. As a result of this shared trauma, the care providers’ thoughts, perceptions, worldview, response to stress, coping, and ability to provide care while experiencing these unique stresses collectively can be impacted. In particular, without time to process and develop coping mechanisms to promote healing and resilience, care providers can experience burnout quicker and easier [35]. Comments to the survey revealed how pharmacy personnel suffered from a shared malaise that came with feelings of being punished, taken advantage of, and used up to the point of utter defeat:

*“The main issue is the whole structure of pharmacy as a retail business with a small number of large corporations controlling the services we provide, how they are provided and how much is made in those service with the profits going to stock holders not the healthcare providers who are providing the services. The expectation is to treat numbers not patients. We are punished when a patient does not take a medication they do not want to take. We are punished when a fellow healthcare professional does not feel comfortable prescribing a medication for a patient for legitimate reasons. We are punished if a patient gets hospitalized and does not take their medication for two weeks. EQUIPP scores are used by PBMs to maximize punishment not reward.”*

*“Chain pharmacy has become nothing more than factory work. Instead of making parts, it’s filling prescriptions as fast as you can all day long. It’s soul killing. Caring for patients? It’s more like caring for their pocketbooks. PBMs add another layer of despicable behavior to their mix.”*

In order to heal from shared trauma, mechanisms on a systems level must be developed and implemented so that individuals can create the time and space necessary to process their relationship to shared trauma and have or maintain the capacity to care for patients in a compassionate manner [35]. Systems interventions that allow for support, time, and training are necessary to help mitigate the harm from shared trauma. While it is important for individuals to work on individual resilience, stress responses and self-care, it is also crucial to critically examine systems and how we treat people in the system.

It is clear that pharmacy personnel collectively know about the shared trauma that they and their patients are experiencing. However, they are suffering and grieving in isolation without any hope of finding a way out. We propose that there is a need for action to identify and prioritize the needs of people within systems rather than seeing and treating them as a means to meet productivity metrics. It is essential to understand their shared trauma and offer functional and healthy systems-level solutions to create time and space for people to thrive.

#### 4.3.2. Threats to Professional Responsibility and Autonomy

Responsibility is a multi-faceted concept with implications for evaluating, sanctioning, and influencing people’s conduct and defined as “the quality or state of being responsible, such as moral, legal, or mental accountability” [36]. In turn, to be responsible carries elements of liability, causality, accountability, and answerability [37].

The extent to which individuals perceive responsibility depends on the psychological connection of three elements: identity images that apply to a person, standards of conduct that should guide a person’s behavior, and events that are relevant to the standards of conduct and the person [38]. Responsibility is judged high when there is a clear, well-defined set of standards for an event, a person is perceived to be bound by the standards by virtue of their identity, and a person has control over the event. In contrast, when standards are ambiguous or conflicting, of questionable pertinence to a person, or the person lacks control over an event, perceptions of responsibility are weakened.

Examples of standards among pharmacy personnel include pharmacy laws and regulations, professional codes of ethics, workplace policies, clinical guidelines, and practice norms among peers [39]. Such standards are internal role manifestations of an external social order, whereby the linkage between standards and a pharmacy personnel’s identity is their sense of professional duty or obligation to these roles that guide how they should act or are expected to act [40]. Control over an event encompasses varying levels that have relative contributions of internal forces, such as foreseeability and intent, and external forces, such as the environment in which an act takes place or in the case of omissions, does not take place [41].

That for which one is responsible and to whom one is responsible are largely dictated by the positions one holds in society. In this context, professional responsibilities of pharmacy personnel encompass a myriad of roles, both traditional and novel. Pharmacy personnel are responsible for and held accountable for roles ranging from dispensing medications to telehealth consultations, patient care decision-making, administering COVID-19 vaccinations, and performing COVID-19 tests [42]. Roles that pharmacy personnel fulfill or perceive they are expected to fulfill include the potential for role overload and role conflict. Role overload occurs when an individual performs multiple roles simultaneously yet lacks the resources to accomplish them, such as time, energy, and capabilities. Role conflict exists when two or more roles are contradictory, incompatible, or mutually exclusive [43]. Role overload and role conflict can lead to diminished perceptions of control and clarity of standards, both of which weaken perceptions of responsibility and autonomy.

Some of the study participants expressed threats to professional responsibility and autonomy that were characterized as disempowerment such as feeling deprived of power, authority, or influence. Several of these comments addressed lack of accountability among organizations that possess power to influence regulatory enforcement, educational accreditation standards, norms, and marketplace dynamics that shape the profession and pharmacy personnel’s working conditions:

*“The boards of pharmacy in every state should be out there visiting pharmacies and seeing what is really happening.”*

*“[The Accreditation Council for Pharmacy Education] needs to stop approving diploma mills and strip accreditation from the worst performing schools.”*

*“Regulatory capture of corporations infiltrating regulatory bodies, educational institutions, and advocacy organizations has effectively emasculated us as a profession.”*

The organizational actions, or inactions, expressed in the above comments magnify the lack of control that these pharmacy personnel have over these issues that are outside of their authority and influence.

The following comments by study participants were indicative of threats to professional responsibility and autonomy via power-dependence. This association implies that if pharmacy personnel commit or professionally invest in resources controlled by corporate pharmacies, they will be dependent on these pharmacies and have less power [44]:

*“Retail pharmacy has the potential to impact patients but are constantly being cut off at the knees with lack of pharmacist overlap and enough support staff making it almost impossible to reach that potential.”*

*“If [corporate pharmacies] truly cared about patients and safely serving them, they would invest in adequate staffing to ensure we do no harm…State boards and state/federal governments must reign in these atrocities for the sakes of patients and the health professionals held responsible for their care.”*

These comments touch upon the notion of the foreseeability to make errors or cause harm. When pharmacy personnel foresee potential harm, they feel responsible for preventing it. However, when they have little or no power to do so, their autonomy to prevent negative outcomes is threatened. Based on these findings, we propose that it would be fruitful to apply human factors and ergonomics systems approaches as outlined by Carayon and Perry [45] for deferring to local expertise, facilitating adaptive behaviors, enhancing system interactions along the patient care journey, re-purposing existing processes, and applying dynamic continuous learning. The goal for such redesigns would be to build resilience in the systems of care so that pharmacy professionals can quickly respond to tensions and disturbances to professional responsibilities.

#### 4.3.3. Learned Subjection

Learned subjection or subjugation results when an organization exerts both high levels of “control” over how work is done and “instrumentality” or the extent to which employees are treated as means toward an end [46]. This category was characterized by comments obtained from pharmacy personnel that represented the elements of oppression, abandonment, depersonalization and dehumanization. Pharmacy personnel expressed feelings of oppression or the inhibition of individuals’ ability to develop and exercise their capacities and express their needs, thoughts and feelings [47] in the following comments:

*“Between the lack of job options and the fear of losing our licenses, corporates/management/leadership figured they can basically increase workload to what it feels at this point indefinitely while they compensate for a set number of hours; I call it the “new slavery.”*

*“Feel like a slave, want to focus on patient care, but that’s the last thing we do if we get lucky.”*

Pharmacy personnel indicated that this type of oppression is characterized by workload that is ever increasing and uncompensated, the inability to focus on patient care, and market forces which lead to a perceived lack of job alternatives. Oppression entails perceptions of powerlessness, marginalization and exploitation [48]. Perceptions such as these may lead to what the nursing literature describes as “oppressed group behaviors” [49]. One such behavior is “silencing the self” which is manifested by not speaking about one’s own contributions to patient care or offering any feedback when asked [50]. These behaviors diminish nurses’ own sense of value and leads to feelings of marginalization, powerlessness and exploitation. Nurses in these situations are often fearful to express their needs which results in low self-esteem. Subjects in our study expressed many of these sentiments.

Feelings of abandonment also were exhibited in pharmacy personnel comments:

*“We are seriously struggling every day and no one seems to care.”*

*“Please do all you can to stop this. Pharmacists for retail pharmacies are literally dying on the job and [COMPANY] doesn’t care. They cannot keep getting away with this and I don’t want anyone else dying just because [LEADER] wants to make more money.”*

*“Yes, we all went into pharmacy to help people. But, when is someone going to help us?”*

Employees develop general perceptions concerning organizational support, which is the perceived extent to which an organization values their contributions and cares about their well-being [51]. Several meta-analyses confirmed that perceived organizational support is positively related to job satisfaction, affective organizational commitment and job performance [52,53]. Research on pharmacy personnel can be extended to investigate the concepts of abandonment and perceived organizational support.

Aspects of depersonalization also were expressed in pharmacy personnel comments:

*“As a pharmacist, I am treated as an easily replacement staff member with no respect for my education level or impact on the community.”*

Depersonalization has been studied in pharmacy as a component of burnout [4,54] and is defined as a dehumanizing response towards people who are the recipient of one’s services. While these studies show that pharmacists experience a high level of depersonalization, the measures used to describe depersonalization refer to pharmacists treating patients in a depersonalized manner and not to how pharmacy personnel feel when they are the recipient of this treatment.

An important aspect of depersonalization is dehumanization. Aspects of dehumanization were found in our analysis:

*“Not only do we not have time to take care of our patients with counseling and providing information with prescriptions as necessary, but we also have given everything of ourselves to try to meet all demands and our own health, mental and physical is waning.”*

*“They show no concern for my personal health at all.”*

*“Corporate does not look at our condition and say what can they do to help, but instead they criticize us for not meeting their goals and that we need to improve on all aspects of their ‘rubric’.”*

Organizational dehumanization is a perception which undermines one’s feeling of having a socially valuable existence [55,56] and arises amongst employees once they realize that they are being considered as a tool or a robot in their organization and can be replaced easily. Many of the comments describing both depersonalization and dehumanization are related to how they feel they are being treated by their employers. One respondent wrote:

*“I feel like I am treated like a robot, with high expectations of completion of all the tasks assigned to us, with many new tasks assigned constantly, but with no additional help given, yet they are constantly taking hours earned away.”*

Research is lacking in pharmacy personnel on these topics and their connection to well-being and resilience.

One final aspect found in the literature related to dehumanization and oppression is violence [57]. In our analysis we found interpersonal violence being perpetuated on pharmacy personnel by patients/customers:

*“Customers have nothing better to do than scream and yell at us and tell us we are incompetent and not fit to work there because of their scripts not being ready when they arrive. Something really needed to be done.”*

Bullying and harassment by customers/patients and co-workers was examined in the 2019 National Pharmacist Workforce Survey. In that analysis about 25% of pharmacists experienced some type of harassment [1]. When workers are frequently abused by their supervisors or others they could feel treated like less than human and could shift these negative perceptions to the organization [57]. Greater understanding of the negative effects of harassment and bullying in pharmacy workplace is needed.

Each of these elements has implications for the pharmacy workplace. Reactions such as these can cause pharmacy personnel to turn inward and suffer in silence. They are also not able to work together to take collective action to better well-being and resilience. This also inhibits the profession in moving forward. Additional work on these aspects is needed. We propose that more research into the aspects of learned subjection by pharmacy personnel including oppression, abandonment, depersonalization and dehumanization would help improve the pharmacy workplace. The findings suggest to us that pharmacy personnel have been mistreated and there is a need for restoration and renewal.

#### 4.3.4. Moral Distress and Moral Injury

Related to the previous section, the results further expose the experience of moral distress and moral injury in pharmacists and pharmacy personnel. One respondent stated,

*“The push to do so much more with less personnel, less trained and/or competent personnel has resulted in a need to come early, work late, work without eating, work without using the restroom…”*

The findings also contained expressions of fear of making ‘very serious mistakes at work’ or ‘worry about maintaining patient safety and providing the best healthcare’ given all of the clinical and technical activities required of pharmacists. Respondents wrote:

*“Giving everything of ourselves to try to meet all demands and our own health, mental and physical health is waning.”*

*“Practice today as “soul-killing”.*

*“I honestly contemplated suicide a few times this last year and no, that is not an exaggeration.”*

Pharmacist suicide must have our attention so that we can take action to deepen our understanding of whether moral injury (or post-traumatic stress disorder) contributes to this alarming outcome as identified in veterans [58,59,60]. It is noteworthy that Lee and colleagues [58] identified a higher suicide rate for pharmacists than the general population during the years of their analysis (2003 to 2018).

Moral distress and moral injury are areas of research for deepening our understanding of what pharmacy personnel experience in an array of patient and non-patient care settings. A conceptual framework has been proposed to illustrate the distinctions and overlap between moral injury and moral distress that may be useful to apply to pharmacy to answer a plethora of questions [61,62]. What factors contribute to the development of moral distress? What impact does moral distress have on the development of mental health issues or on the development of moral injury? Are there differences seen amongst those engaged in different direct patient care settings and non-patient care settings such as working in regulatory or medical affairs within a pharmaceutical company or making decisions pertaining to managing populations? Further, applying moral distress research to pharmacy can help inform what organizations and regulatory bodies need to do to enrich the work environment to enable pharmacy personnel to meet their responsibilities to their patients and communities.

Moral injury currently lacks consensus around a definition and literature in this area is in an early adolescence stage—especially in pharmacy. Identifying and adopting a conceptual framework that is applicable across different areas of the profession and integrating assessment as part of future pharmacy workforce studies would be useful for characterizing moral injury experienced by pharmacists. A recent critical literature review of moral injury proposes research needs that may have applicability to pharmacy such as determining the relationship between specific morally injurious events and outcomes; broadening examination of moral injury to pharmacy personnel; or considering how moral injury may amplify the risk for developing substance use disorders or exacerbating them [63]. It seems logical that moral injury contributes to suicidal ideation or that worry over committing medication errors leads to anxiety. However, it is necessary to discover whether specific associations exist. Pharmacy-specific research on moral injury is critical to learn how moral injury develops for pharmacy personnel. The determination of effective strategies for managing it and how to pressure-proof pharmacy personnel during their careers to mitigate its onset is essential.

#### 4.3.5. Sociocultural Effects

The findings affirmed that sociocultural effects, such as workism, are part of how pharmacy personnel describe their workplace wellbeing and resilience. Respondents wrote:

*“Please help, I can’t believe my career has come to this. I’ve become obsessed with work only to have it take over my life.”*

*“Help us. We are drowning. We are dancing as fast as we can, but somehow the platform gets wider and wider, our shoes smaller, and the music louder. No one can keep this up.”*

The sociocultural phenomenon of workism is an area of research for expanding our understanding of pharmacy workplace wellbeing and resilience. There is a need to develop innovative ideas for helping our society, including health care systems, evolve and mature in response to the need to help health care providers experience self-actualization outside of salaried jobs [22,23].

Another area for future inquiry that is related to workism is how large-scale reform can improve the experience of providing and receiving care. Halfon and colleagues [63] propose that there is need to integrate societal and health care system interests into the model of health care. That is, there is a need to advance from a coordinated health care system in which the focus on making the system more efficient, effective, and profitable to one that is community-integrated. Together, health care providers and patients can build community-integrated health systems that meet the needs of specific communities and use a bottom-up approach for developing systems of health optimization. This could be a way to overcome workism and the stress that comes from feeling trapped in one’s work.

Finally, we propose that including the quintuple aim for health care improvement is needed. The quadruple aim—improving population health, enhancing the care experience, reducing cost, and improving the experience of providing care—was enhanced in 2022 by Nundy and colleagues [64] with the pursuit of health equity being elevated as the fifth aim for health care. We propose that this sociocultural shift will be important for improving wellbeing and resilience in health care for both providers and for patients. Building the opportunity to attain one’s full health potential with no one disadvantaged from achieving this because of social position or other socially determined circumstances will change incentives from top-down, mechanized, assembly line processes back to incentives that reward intimate caregiving relationships.

We propose that such change is possible as patient-tailored virtual communication, genomic-customized treatments, collaborative engagement for patients as co-producers of value, and anticipation of needs already are being driven by technological advancements [65]. Research in the areas just presented will be fruitful and will help drive positive change for improving both patient and practitioner wellbeing.

#### 4.3.6. Plea for Health Systems Change

The previous sections have discussed the state of professional well-being for pharmacy personnel and described determinants of burnout and career satisfaction [66,67]. Evidence from respondents’ written comments suggests that individual-level interventions are inadequate for addressing the endemic issue of burnout in pharmacists [5,66,67], a gap that is perhaps best described by this quote from a 2018 article by Gregory and colleagues: “*Interventions to reduce burnout have sought to improve the resilience of an individual to withstand this [job demand–job resource] imbalance rather than identify and ameliorate the cause*” [68]. This realization informs the need for pharmacy professionals to embrace a systems orientation in their efforts to improve well-being in the pharmacy workspace. A systems framework conceptualizes pharmacy work environments as complex systems in which the resources, constraints, incentives, and demands interact. Organizations influence how employees engage with their work within the organization as well as the individual and organizational outcomes derived from work [69]. Three areas constitute the focus of best practices for a systems approach to improving pharmacy professional well-being [69,70]: (1) aligning professional and business roles, (2) work-system redesign and organizational learning, and (3) rethinking legislative and regulatory frameworks.

The pharmacy profession, especially in the community setting, has been struggling to settle the conflict between professional and business roles for over a century [71]. As mentioned previously, role conflicts are a source of dissonance that pharmacists experience at work. Corporate goals of efficiency and profit maximization and pharmacy professionals’ goals of patient care often do not align [72]. One respondent wrote:

*“12 years ago I was in a slow store and was greatly able to make an impact on my patients and their health. However, in more recent years I have been required to increase sales, profit, script count…”*

Studies have shown that when corporate and professional values align, health care professionals have a more positive view of company culture. This has been associated with improved work engagement, performance, and satisfaction among healthcare professionals [72,73]. Addressing role conflicts in pharmacy organizations requires all stakeholders to recognize the benefits of pharmacy professionals’ work in an effort to provide better care to patients and help them engage in work that aligns with their professional interests. Pharmacy organizations will benefit from finding common ground between process efficiency and profit maximization goals with pharmacy professionals’ need for joy and fulfillment at work [74]. This includes redesigning organizational structures, processes, and policies to prioritize meaningful work for personnel even if it may come at short term financial costs to the organization through the need for more resources and making job demands more in line with pharmacy personnel operating in a professional orientation. The long-term benefits are expected to include employee retention and better quality care, which will be important for pharmacy to continue to move toward being reimbursed for providing value to health systems.

As alluded to in the previous paragraph, redesigning organizational work processes and systems begins with improving the nature of the work that pharmacy professionals do. Excessive workload, monotonous and low-value work that do not require personnel to use their skills and training to the fullest extent are major contributors to burnout [5,75]. One respondent wrote:

*“Pharmacists are doing the best they can but the workload we are juggling is an error waiting to happen.”*

Physical aspects of pharmacy workplaces need to be redesigned by incorporating principles from human factors, ergonomics, and human-centered design to reduce occupational stress and fatigue [76,77]. Qualitatively, balance must be maintained between job demands like workload, length of work shifts, and job resources like organizational support, teamwork, and appropriate staffing [5,78]. Organizations have a duty to involve pharmacy professionals in decision making, development of performance metrics, and performance evaluations in a feedback loop that fosters organizational learning. Research shows that such a collaborative approach reduces the chasm between management and staff, as the staff are more likely to view leadership as authentic [79].

While pharmacy professionals and organizations may not be lacking in intent to implement professional well-being systems at work, it appears that such efforts often fall short of their objectives without effective legislative and regulatory frameworks. It was the view of many respondents in the present study that role conflict and work stress persist due to a lack of external mechanisms to hold pharmacy organizations accountable. One respondent wrote:

*“Pharmacy chains are promoting dangerous workplaces, both for the patients and employees. I sincerely hope state legislators or federal regulators step in before there is some horrible tragedy.”*

There is already enough tragedy in the work life of pharmacy professionals to warrant urgent legislative and regulatory interventions. Areas of pharmacy work life that can be targeted for regulatory intervention include ensuring effort-reward balance to deter performance metrics and compensation schemes that encourage quantity over quality of work done [80]. To a greater extent, there is a need for effective alignment of incentives to drive the desired system changes. Pharmacy professionals have noted that while the managerial pharmacist-in-charge (PIC) may be the representative of a pharmacy organization, as one respondent put it:

*“The PIC is responsible for everything but has no control over anything such as training, staffing, hours, budget… yet the boards go after the PIC.”*

Additionally, pharmacy professionals and organizations need better representation in legislation making. The future pharmacy workforce should also be educated about the challenges in pharmacy workplaces with a view to identifying opportunities for improvement.

## 5. Conclusions

There is shared malaise surrounding a collective experience of helplessness, loss of professional standing, oppression, moral injury, workism, and a plea for rescue. The results show the need for health care system and societal change and provide insights for future research.

We propose that there is a need for more expansive and extensive research that is specific to pharmacy workplace wellbeing and resilience. One area we uncovered in this study relates to the construct of “shared trauma.” Tosone and colleagues [35] outlined terms such as burnout, compassion fatigue, secondary traumatic stress, vicarious trauma, and counter-transference. These resonate with our findings and merit further study for how systems can be changed to help pharmacy professionals have or maintain the capacity to care for patients in a compassionate manner.

Another domain for inquiry relates to “professional responsibility and autonomy” for assuring patient care and medication safety. We propose that human factors and ergonomics systems approaches [45] would serve as useful foundations upon which to base further research. Carayon and Perry [45] outlined the importance of systems that are deferential to local expertise, adaptable, interactive, based on existing processes, and dynamic with continuous learning. Such systems would improve professional responsibility and autonomy for patient care and medication safety.

“Learned subjection” was another theme that emerged in the findings. Several research domains were identified that could be useful for future work including: social justice [46,47,48], challenges observed in nursing [49,50], organizational support [51,52,53], and psychology [55,56,57]. In a related manner, “moral distress and moral injury” research is rooted in the psychology literature as well [58,59,60,61,62]. Learning more about possible links among learned subjection, moral distress, moral injury, anxiety, suicide, medication errors, and substance use disorders is necessary. Relatively little is known about these in the pharmacy domain. Based on the findings from this study, we propose that these topics deserve more attention.

In addition to research that is focused on individuals, more work is merited for “sociocultural effects.” Our findings revealed that the sociocultural phenomenon of workism [22,23] is negatively affecting pharmacy workplace wellbeing and resilience. Activities focused on building community-integrated health systems that meet the needs of specific communities and applying a bottom-up approach for developing systems of health optimization could be helpful. Halfon and colleagues [63] proposed that there is a need to integrate societal and health system interests into a new model of health care. We propose that such a sociocultural shift would improve the wellbeing and resilience for both providers and for patients.

The findings showed the need for “health systems change,” another form of systemic support. Current systems are inhumane in that they are creating burnout [68]. Hence, we propose the application of a systems framework [69,70] in pharmacy. Some work has been reported in the physician and nurse domains [68,69,77] and application in pharmacy is justified. Specifically, there is a need for (1) aligning professional and business roles, (2) work-system redesign and organizational learning, and (3) rethinking legislative and regulatory frameworks [69,70].

In summary, business models driven by mechanized assembly line processes, business metrics that supersede patient outcomes, and reduced pharmacy professional judgement have contributed to the decline in the experience of providing patient care in today’s health systems. The results showed that the portrait of respondents’ lived experiences regarding pharmacy workplace wellbeing and resilience is beyond the individual level. We propose several areas for expanded inquiry in this domain: (1) shared trauma, (2) professional responsibility and autonomy, (3) learned subjection, (4) moral injury and moral distress, (5) sociocultural effects, and (6) health systems change.

## Data Availability

Data used for this study is stored in electronic format and may be obtained from the corresponding author at schom010@umn.edu.

## Appendix A

**Table A1:** Elements and Exemplars from Hermeneutic Phenomenological Analysis.

Shared Trauma	
Element	Exemplars from Written Text
Helplessness [lacking protection or support; inability to act or react]	[6601.00] The main issue is the whole structure of pharmacy as a retail business with a small number of large corporations controlling the services we provide, how they are provided and how much is made in those service with the profits going to stock holders not the healthcare providers who are providing the services. The expectation is to treat numbers not patients. We are punished when a patient does not take a medication they do not want to take. We are punished when a fellow healthcare professional does not feel comfortable prescribing a medication for a patient for legitimate reasons. We are punished if a patient gets hospitalized and does not take their medication for two weeks. EQUIPP scores are used by PBMs to maximize punishment not reward. We should have a reimbursement model the same as other healthcare providers have through Medicare. Start off at a base pay. Then rewarded for hard work. The current PBM systems starts off all in a hole butting pressure of bosses to sell, sell, sell not do what is in the best interest of the patient. [3750.00] Chronic understaffing has been a nightmare. I graduated in 2021 from college and am currently the only regular pharmacist working at a store that fills 250+ prescriptions a day and administers 42+ vaccines per day. I am severely undertrained for my job, but everyone with more experience left. It has been mentally draining. [3742.00] Most of our non-24 h stores have only 2 pharmacists on a weekday. Some stores that are busier really need a 3rd midshift pharmacist. Unfortunately, corporate keeps cutting out budget. There’s nothing that we as employees can do.
De-professionalization of Pharmacy	[6489.00] Ridiculous documentation is required to fill controlled substance prescriptions. Constantly questioned, threatened, and berated about our professional judgement. We are expected to write a “story” to justify filling control substance prescriptions. This is very time consuming and a distraction from patient care. I would never fill a prescription that I didn’t believe was being used for a legitimate medical reason! Stop forcing us to police the prescribers. I wasn’t in the exam room, operating room, hospital room or shown the test results. I don’t know how many MME are required for major orthopedic procedures or diverticulitis. I don’t think I should be calling and questioning what is adequate for pain coverage for a patient who is sent home the same day of major surgery. We’re reporting to our respective state data bases. If the board of pharmacy or medicine thinks the doctor is over prescribing, go after the doctor. I had open heart surgery and was sent home with tramadol because the physicians are so afraid to prescribe opiates. I was miserable for two weeks. I was in so much pain that I barely slept. Don’t punish legitimate patients because of people who aren’t legitimate. [6311.00] Chain pharmacy has become nothing more than factory work. Instead of making parts, it’s filling prescriptions as fast as you can all day long. It’s soul killing. Caring for patients? It’s more like caring for their pocketbooks. PBMs add another layer of despicable behavior to their mix. [4219.00] Our profession has unfortunately gone WAY downhill. Job security is at an all-time low. Retail is becoming more and more like glorified cashiers. New students cannot find jobs. Part time jobs are only scheduler for 4 h at a time. Corporate America does not care about the person, the more you move like a robot the happier corporate is… [5083.00] Insurance companies, PBM’s, state boards (conflict of interests) have legitimately ruined pharmacy… they have taken a once respected profession and turned it into a mere factory. Patients have no clue how hard/over worked pharmacy staff are…how educated pharmacists are. Honestly retail pharmacy (outside of okay wage which is decreasing) is one of the worst jobs. I never do surveys but honestly pharmacy as a profession is a GD JOKE! Thanks corporation’s/PBM.
**Threats to Professional Responsibility and Autonomy**	
**Element**	**Exemplars from Written Text**
Disempowerment [to deprive of power, authority, or influence]	[6835.00] The boards of pharmacy in every state should be out there visiting pharmacies and seeing what is really happening. We as pharmacist feel we can’t complain. We need our jobs and can be replaced with new grads at a huge savings for the company. I have kids that need to eat and have a place to live so there is no way I can do anything about this problem. We need a movement. [3322.00] My job was jeopardizing my sanity and so I had my husband buy a house a few hours away so I could come up with an amicable reason to leave. I had no plan and didn’t care. This is my last week of working at that store. I was a bright, career driven pharmacist turned cynical and depressed in less than 5 years into my career. Burnout is real and if the industry isn’t careful most will leave in the next few years. [3448.00] ACPE needs to stop approving diploma mills and strip accreditation from the worst performing schools. The glut of new, poorly trained pharmacists continues to weaken the profession and continues to cause working conditions to decline. The profession doesn’t have the ability to negotiate from a place of power for improvements to patient care if we’re this diluted. [4035.00] Regulatory capture of corporations infiltrating regulatory bodies, educational institutions, and advocacy organizations has effectively emasculated us as a profession. Scope of practice needs to dramatically increase along with reimbursement models that allow pharmacists to break free from traditional roles and grow alternative work models and foster entrepreneurship, otherwise we will all be slaves for these corporations that will eventually ruin the profession.
Power-dependence “The more a person values resources controlled by another, the more dependent that person is and the less power he/she has in the relationship.” [44]	[6437.00] There is no loyalty to any workers anymore. The company asks, what have you done for me today? You were paid for yesterday. So if you ignore your own family working 12-h shifts and come home to an empty house, they like that, because they become your family and your only life and can treat you however they like and you will not quit. [3118.00] Retail pharmacy has the potential to impact patients but are constantly being cut off at the knees with lack of pharmacist overlap and enough support staff making it almost impossible to reach that potential. [6638.00] Unfortunately, corporate pharmacies have been allowed to become an entity that fears nothing. They talk a good game, but [they are] in it only for the money. If they truly cared about patients and safely serving them, they would invest in adequate staffing to insure we do no harm. Pharmacists are being asked to sail aircraft carriers single handily with no crew onboard. State boards and state/federal governments must reign in these atrocities for the sakes of patients and the health professionals held responsible for their care. [5478.00] Most pharmacists I talk to work many hours off the clock to keep they’re pharmacies from falling behind, my employer even said this is expected and is the new normal. Working 14 or 15 h on a 12 shift. Most pharmacy managers have to work on their days off to get the day-to-day projects done as there is not enough payroll given to do in workflow. (Again this is the expectation, not the exception) [3839.00] Pharmacy needs to be treated with more value not held accountable for the stores profits, allowing more staffing and being able to close for lunch. The retail/grocery side should not make more than the pharmacists especially since the lack the education we go through. The retail/grocery side should not be able to tell the pharmacy what to do. They have no knowledge of the laws.
**Learned Subjection**	
**Element**	**Exemplars from Written Text**
Oppression [burdened by abuse of power or authority]	[2790.00] Unless state boards decide to intervene to regulate required minimum staffing in proportion to number of prescriptions/vaccines done daily, nothing will change. Corporates know we cannot get other jobs; they also know we work under the privilege of our license, and WE (as pharmacists; not them!) are the ones responsible for what we do or do not do in the pharmacy. Between the lack of job options and the fear of losing our licenses, corporates/management/leadership figured they can basically increase workload to what it feels at this point indefinitely while they compensate for a set number of hours; I call it the “new slavery.” My biggest fear as a fairly new practitioner is that as I feel literally exhausted without even getting a meal/rest in so many hours, I will not be able to maintain minimum professional standards, I will make a bad mistake, or I will not have a life sustaining medication ready, and my patients will suffer… or die. [5594.00] Administration is retaliating against me because they say that I have disrespected them but they gave me no respect from the day that I started and tried to punish me and threaten my job because I spoke out about things that needed changed in the pharmacy and they have no idea what goes on in a pharmacy and do not want to learn even though I have asked if I could show them. They said they didn’t have time. [4713.00] My job as a retail pharmacist is hell, I would never ever want my child go to a pharmacy school and spend roughly 200 k to do this job. We at least used to get paid well, not anymore. Feel like a slave, want to focus on patient care, but that’s the last thing we do if we get lucky. It’s not easy to catch errors and get prescriptions out without any error while being rushed by corporate and customers and then get yelled at from both sides [5048.00] Corporate retail pharmacy is currently a dumpster fire. Pharmacist and technician burnout is the highest I’ve ever seen and financial obligations are currently the only reason I continue to stay employed as a pharmacist. [5567.00] I left retail pharmacy after practicing for 20 years. I felt forced to change my occupation since every retail chain outlet had left staffing so low that the stress level for everyone was out of control. I had nightmares after I worked each day that I might have made a mistake since I had to go so fast between ringing phones, drive through window, and pick up window with little or no breaks. When staff would bring up to management about the unsafe work conditions, they would do nothing about it. Management also encouraged staff members to work sick to show you were tough. You were made to feel bad that you had to call off. During HINI outbreak in 2014, I came down with flu symptoms and went to work anyways. I made an error that day I was sick. I didn’t catch an expired insulin that went to patient, which resulted in citation by the Ohio Board and Michigan pharmacy board. I was crushed beyond measure, to be punished for mistake that a patient didn’t even inject or use. I went to work sick, which I should not have done, but my employer did not give sick days and gave employees negative attitude if you did call off. When the potential for a fine came about, my employer told me they would pay any fine that the board would impose. When I did get the fine, my employer changed their mind and, told me sorry, they would not pay it. I felt betrayed by my employer and the board of pharmacy who didn’t even address WHY the mistake happened. Mistakes are a chain of failures. In the end, I found another job in regulation, where I am valued, have enough sick leave that I can use and not have to justify it, and am respected by both customers and employees. I will NEVER go back to retail pharmacy, and if anyone asks me why I tell them it is a profession that has changed for the worse and you will be punished for things that you cannot control.
Abandonment	[5951.00] We are seriously struggling every day and no one seems to care. Unfortunately it’s going to take a mistake that harms a patient (and gets blamed on the stressed out, overworked pharmacist) to put a stop to this madness. [3376.00] Things need to change for all pharmacists and techs in all settings. Our practice is unsafe and no one cares until after a patient is negatively affected. Too much on everyone’s plates. Pharmacy is not a great profession anymore for mental, physical, and social health. [4332.00] Just more awareness that we are all burned out on healthcare and need more support. Other companies are worried about employees well-being during a pandemic, but I do not feel that my company cares about my mental health and stress during this unprecedented time. [3694.00] Please do all you can to stop this. Pharmacists for retail pharmacies are literally dying on the job and [COMPANY] doesn’t care. They cannot keep getting away with this and I don’t want anyone else dying just because [LEADER] wants to make more money. [6087.00] The pressure of being the most accessible healthcare provider is getting too much for us to handle. Yes, we all went into pharmacy to help people. But, when is someone going to help us? Between, tech hour cuts, lack of techs, lack of tech pay, pharmacists are doing their job plus often, the techs job too. How does corporate think a single human being can be cashier, tech, pharmacist and vaccinator all at the same time? But they don’t care about the well-being of their employees. All they care about is the money! I hope the money helps them sleep at night when hundreds of thousands of patient’s lives are at risk everyday because their pharmacists are overworked, stressed out, hungry and being pulled in 100 directions at once. [4765.00] The profession of pharmacy has endured much over the years in silence because we believed that patient care trumps all else. Now we are all burnt out and asking for support, being vocal for the first time as a profession, and being met with silence. It is pervasive and everywhere in every field of pharmacy. We are underappreciated, taken for granted, and now when our physical health is being impacted, we are saying no more. There’s a reason retail pharmacies are shortening hours or closing early all over the country, incentives or not. Please help us be heard.
Depersonalization (Robots; loss of sense of identity)	[3895.00] The COVID immunizations have shown the greed of major corporations. They are pushing pharmacists beyond their limits. Days off are being denied because of lack of pharmacists due to turnover. We are being exploited as pharmacists and being used as robots. The expectations to answer a phone in a certain time, complete patient adherence calls twice a day to reach a certain percentage of people, complete MTM, all why providing patient safety and good customer service is next to impossible. [6371.00] As a pharmacist, I am treated as an easily replacement staff member with no respect for my education level or impact on the community. More and more tacks are added to my plate without an increase in staff or a worry about safety of my patients [6635.00] I feel like I am treated like a robot, with high expectations of completion of all the tasks assigned to us, with many new tasks assigned constantly, but with no additional help given, yet they are constantly taking hours earned away. They show no concern for my personal health at all. I don’t get any designated breaks or lunch/dinner. It is frequently difficult to go to the bathroom or eat. I am stressed because I can’t get everything done that is expected. I don’t want more pay. I want reasonable breaks and more help. I worry greatly about my health as a result of this!!!
Dehumanization	[4535.00] The push to do so much more with less personnel, less trained and/or competent personnel has resulted in a need to come early, work late, work without eating, work without using the restroom, etc. to where pharmacist health is last priority. Not only do we not have time to take care of our patients with counseling and providing information with prescriptions as necessary, but we also have given everything of ourselves to try to meet all demands and our own health, mental and physical is waning. It’s hard to pour from an empty cup. Once those who care leave, who will take care of patients and make sure to keep them safe?? [4238.00] Things have to change. Patient safety is severely at risk due to corporate greed. All of us in retail pharmacy are severely burn out and our mental and physical health have taken a toll. Lunch breaks and bathroom breaks should not actually be considered job perks, but they are. Taking a 5 min breather should not be unreasonable. No job should be giving its employees suicidal thoughts. Cooperate greed has ruined what should be a great career and it is putting the lives of patients at risk. No one should go to work praying they do not accidently hurt someone. Something has to change. [5575.00] Pharmacists are treated more and more, as each year passes, as disposable and robotic. We are not treated humanely or given proper rest time. We are expected to work 8–13 h shifts, standing the entire time, with one 15 min break. This is terrible and no one should be treated this way. It’s all about how productive you are, non-stop. No time for relaxing is allowed at all which seems completely unacceptable. No matter what the profession is, people need a chance to digress and get away from the pressure more than 15 min per day! [4289.00] I have worked 17 h straight (and only got paid for 12 h) with no breaks because the pharmacy has an inadequate amount of staff to keep up with the work. All my techs stay late past their scheduled hours and we still can’t keep up. The scheduling with no lunch breaks or any break at all is brutal. Again, I have worked 12–17 h with NO meal breaks. I have to stuff food down my throat or have liquid lunches in order to stop my hunger. I feel guilty when I have to take a bathroom break, which is ridiculous. I am exhausted and ready to quit this profession that would treats its workers like garbage. [3566.00] My pharmacy and several other pharmacies are sinking, we are all so over worked and understaffed. Every day I walk into work with someone calling out, someone quit or someone didn’t show up to a job interview. The fill is always over 300 and every customer is screaming and yelling at us to get their meds to them right away. With the short staff we have, along with only one pharmacist per shift, and over 60 shots each day, working at [COMPANY] has degraded all of our mental health and we are physically and mentally exhausted. Corporate does not look at our condition and say what can they do to help, but instead they criticize us for not meeting their goals and that we need to improve on all aspects of their “rubric”. This is inhumane as to how bad the working conditions have become. My coworkers and I have not taken a break in years because of how busy we are. There is no time to eat or use the bathroom, let alone take a break. The pharmacist are so hard working but even they are not allocated a break. My store is 24-h and every store in our district is given a half hour break except us. We are open every holiday, we never closed throughout the pandemic. We hire more technicians and they all leave due to lack of proper training and just how bad the work conditions are. My store gave people a 1200 dollar bonus to come work for [COMPANY] and even after that people did not want to work after seeing the working conditions. There is something seriously wrong. We are all so over worked and exhausted. We have to come in extra every day because we have no other choice. Every day we work out asses off and yet everyone is unhappy and it seems as if no works get done. Our fill stays at 300–500 scripts everyday, we clean it up and the next day the fill is worse than before. Customers have nothing better to do than scream and yell at us and tell us we are incompetent and not fit to work there because of their scripts not being ready when they arrive. Something really needed to be done.
**Moral Distress and Moral Injury**	
**Element**	**Exemplars from Written Text**
Moral Distress “Negative feeling that follows when clinicians believe they know the morally best action to take, but a different action is taken, often for a variety of reasons”. Dissonance experienced when people are not able to offer care in the way that is best/optimal. Ulrich, C. M. and C. Grady. 2019. Moral Distress and Moral Strength Among Clinicians in Health Care Systems: A Call for Research. *NAM Perspectives.* Commentary, National Academy of Medicine, Washington, DC. https://doi.org/10.31478/201909c, accessed on 5 November 2022	[5882.00] We are doing more work with less help. I have been with my employer for 21 years. I feel they are one of the better retail sites but it is still not great. We hardly get raises, in fact, they cut our hours of operation and cut staff and floaters to 30 h. So all the same work needs to get done, with ten less rph hours per week. The stores run on a skeleton crew with little chance of a filling a call out. The pay for techs is not competitive enough to keep anyone for long. The days feel like a rollercoaster ride that has started before the rider is properly strapped in. The day is just holding onto the bar and trying to survive. AND all this while having to be professional and composed. We do not get breaks. We eat on the go, we work when we are not feeling well or are sick. Also we are asked to set up clinics for flu shots on our own time. And the public behaves poorly. Tension in the world is high and sometimes situations escalate and we do not have training on how to handle it. [3113.00] If we let [COMPANY] continue to treat their pharmacists the way they do it will be the death of retail pharmacy. Technicians cannot replace pharmacists. The stress of the job is so high that we hemorrhage good technicians and often just cycle through techs all year long retraining only for them to realize how stressful it is and leave. The idea of virtual verification being implemented and the tech doing everything is such a danger to patients in this environment. Techs are not adequately trained you can ask any tech at [COMPANY]. It’s a serious danger to patients and I worry about the amount of mistakes that will be made. As [COMPANY] you are not just a pharmacist, you are pushing metrics all day, immunizing, you are the IT department, you are HR. If you’re severely understaffed with techs and behind they do not care, it’s your problem as long as you’re there to keep the pharmacy open it doesn’t matter to them. [COMPANY] is absolutely destroying community pharmacy. [4822.00] Retail pharmacy is BROKEN. I love my job and profession, but feel so much pressure to “keep up”, I sometimes feel as though we are a danger to our patients. We’ve set this 15 min or less expectation and both patients and employers don’t seem to understand that speed shouldn’t be the priority. [4733.00] The state of retail pharmacy is dire. If retail pharmacists could ban together to strike it would hugely benefit us, but how selfish to close and essential healthcare site for our patients! Hard to find a retail pharmacist willing to do that to their patients.
Moral Injury In the context of trauma or stressful situations, the engagement in, failure to prevent, or witnessing events contrary to held (moral) values/expectations. Can occur outside of military context. Symptoms can include range from grief, guilt, and despair. https://www.ptsd.va.gov/professional/treat/cooccurring/moral_injury.asp, accessed on 5 November 2022.	[3157.00] At what point am I able to say no more? Every day in a retail pharmacy is like trying to drink from a firehose. It is too much. But I am a professional. So I can’t get overwhelmed. I can’t turn away customers and 6 more vaccines that want to wait in front of the other 6 people already waiting when I’m 72 h behind on prescriptions and getting yelled at left and right, I can’t not answer the phone, I can’t step away for any part of a second. But I want to. My hands are shaking. But I can’t let them see that. No one person could keep up with the amount of prescription filling, question asking, answering, vaccine giving, insurance explaining, and let me build a relationship with you while I’m at it, make sure you trust me, and help you understand this new life changing diagnosis you just received. Yea right. We need help. We don’t have adequate technician help at any time and it’s too much for one pharmacist. The pharmacist never leaves their shift on time, we come in early, stay late, off the clock, working well over the state mandated allowable amount to try to lighten the load they will return to the next day. But it never gets better. The idea of a “lunch” break is comical. It’s not physically possible the way the pharmacy operates. I eat as fast as I can while answering questions and pretending I’m not working the entire time. Making the company keep a log book does nothing except making me waste more of my precious time to write something down that didn’t happen. If I step away for a moment everything stops, piles up, and gets even worse and I can hear my one tech getting yelled at because the doctor called it in this morning and why isn’t it ready yet so I just come do it. I can’t think critically about any one thing at any given time when I’m being pulled 87 directions at once and every person thinks they are more important than every other. We need more help. [5365.00] I have multiple calls from other pharmacist that are now on antidepressants due to the work place stress that is forced upon us. We are all afraid of retaliation for negative comments toward our management staff that we don’t make them because we need the job to pay our bills and provide for our family. I’m sure 90% would change jobs if they could. I pray insurance companies are put in check, budgets can be reevaluated and things put back into the way it should be.
**Sociocultural Effects**	
**Element**	**Exemplars from Written Text**
Despair	[5886.00] Help us. We are drowning. We are dancing as fast as we can but somehow the platform gets wider and wider and our shoes smaller and the music louder. No one can keep this up. [5072.00] Corporate chain pharmacies are absolutely destroying this profession, especially over the course of the last year. I had a tech call out one day and worked the entire day by myself filling prescriptions, giving COVID vaccines, and performing COVID tests in drive thru at 10-min intervals. I texted my boss and his response was that we can’t stop any of the services for the day just because we don’t have staff. I stressed that this was unsafe and he shrugged it off. That was the moment I realized I needed to leave this position and thankfully I am in a few days. Let’s not even get into the mental and physical strains this has put on me as a pharmacist and person. I honestly contemplated suicide a few times this last year and no, that is not an exaggeration. [6046.00] Please help, I can’t believe my career has come to this. I’ve become obsessed with work only to have it take over my life more than all the free time I have already given. [4774.00] [COMPANIES] should have millions of fines due to the unsafe situations they have put their staff in. They keep making the work more miserable so more seasoned staff quit and they can replacement with cheaper help like $35/hr. In order to get everything done, pharmacists are working tons of extra, unpaid hours to compensate. They say we’re “salaried” but if you miss a shift, they dock your pay or use pto. There’s no way to leave in the middle of the shift to pick up a sick kid from school or get an ambulance while having a heart attack because there’s no one there to run the pharmacy if a pharmacist isn’t present and there’s never any overlap. So you get none of the perks of being salaried. I’ve attempted suicide due to the unsafe working conditions and no hope for improvement. These chains have ruined the progression of Pharmacy and have ruined many pharmacists along the way.
Disdain	[3562.00] Working at [COMPANY] is horrible. Rx errors because of how insane busy we are. No time to do anything. Work like a literal dog for noncompetitive wages. Then [COMPANY] asks why can’t we find anyone to work. Because you can go to McDonald’s and make more money. It’s insane.
**Plea for Health Systems Change**	
**Element**	**Exemplars from Written Text**
Different Cultures (Professional vs. Business)	[6967.00] Upper management needs to take care of their employees FIRST. Help change customer perception from a “fast food” expectation for pharmacy services——right here, my way, right now. Put some accountability back on the patients—-it’s not the pharmacy’s job to remind the patient of everything—-refill due, your RX needs refills, your RX is ready, your RX needs a prior authorization, your RX has been here ready x 5 days, etc. We don’t have time to be every patient’s keeper. [5884.00] I have spent 24 years in pharmacy and the decline that started slowly in 2010 is speeding towards a cliff. Corporate has lost sight of the goal of pharmacy practice to provide the right meds to the right person in the right dosages for a positive outcome. Asking pharmacists to police opioids, provide vaccinations, basic screenings, advise otc recommendations, take phone calls for all areas of a store are draining the life out of quality pharmacists. [4619.00] I love my job as a pharmacist and I love helping my patients. When I first started pharmacy as a pharmacist 12 years ago I was in a slow store and was greatly able to make an impact on my patients and their health. However in more recent years I have been required to increase sales, profit, script count, understand and know my P&L without any training, become a marketer for IMZ, scripts, specialty without help/guidance on my own time without help from the company only to name a few requirements. Unfortunately all of these requirements have decreased my time to interact and actually help my patients. [4697.00] Please help hold [COMPANIES] accountable. These corporate giants could care less about its pharmacy staff, let alone patient safety and instead they only worry about profits. I have witnessed 6 pharmacists quit in my area, 1 go to part time and numerous techs quit as well due to this hostile work environment. Pharmacists are doing the best we can but the workload we are juggling is an error waiting to happen. We are told flu +2 shots is an expectation daily (COVID shots don’t count in this metric). We are told skip lunch if you need to. We are told daily how new grads make less money and would die to have our job. When we ask for more help, they tell us that’s not an excuse to not meet all metrics. This is dangerous and I worry about my patients as well as my own health. #pizzaisnotworking
Now is the Time	[3159.00] Pharmacists have far more power than they realize. It is time to stop forsaking our profession and patient care for fear of our jobs. Quality of job should be a focus, mental health of our colleagues should be a focus, strong consideration of a Pharmacist Bill of Rights should be developed. We need to stand together to improve our day to day conditions.
Plea for Change	[6754.00] The boards need to step in and mandate staffing or at least fine/sanction the stores licenses and quit including the PIC, because they have no control over any of this. The Pic is not going to complain if his license is always on the line… And they all know it… The PIC is responsible for everything but has no control over anything, such as training, staffing, hours, budget… yet the boards go after the PIC. [4020.00] Pharmacy chains are promoting dangerous workplaces, both for the patients & employees. I sincerely hope state legislatures or federal regulators step in before there is some horrible tragedy. [3912.00] Regulations are needed to ensure adequate staffing and breaks for pharmacy staff. The constant understaffing is leading to significant burnout and mental health issues. PBMs also need to be regulated to prevent limiting patients access to quality care. [3143.00] There should be rules and policies in place to prevent corporations from putting profit before patient safety and well-being. I spend 98% of my time at work doing tasks to keep my performance metrics high in order to please my non-pharmacist boss. I have to rush thru my clinical decisions or simply override them to have enough time to help my understaffed team with non-clinical tasks. I keep my liability insurance updated because I know with the speed, multitasking and constant interruptions that I’m expected to work making errors is inevitable and my employer doesn’t care at all. [6590.00] Please put in legislation that stops DIR fees and any loopholes for PBMs and insurance companies to keep profits out of pharmacies. We are NOT being greedy. We are trying to survive. They continually invent new ways to audit us and the amount of nitpicky documentation that we have to do to avoid audits is ridiculous! [5632.00] Poor reimbursement and DIR fees have been the main cause of the problems in pharmacy. My husband is a pharmacist for a chain and I work for an independent. The chain has cut back on staff so bad that my husband who used to love his job now dreads going to work because the understaffing makes it dangerous to perform his job safely. They have virtually no pharmacist crossover which means he has days when he works 12 h with one 30-min break. Every pharmacist duty falls on him. When you are being pulled in 25 different direct because there aren’t enough workers, it leads to a high probability for errors (which can be dangerous in the pharmacy). In my case, working for an independent, my boss knows the dangers of understaffing and therefore we have 2 pharmacists during most working hours, but I see firsthand how insurance reimbursement rates are destroying our business. Something needs to be done about this problem before good reliable independent pharmacies like ours are forced to close. [6477.00] State laws need to be enacted that hold upper-level management responsible for the decision they make that negatively impact patient safety.
